# NARVGA: A Hybrid Framework Integrating Matrix Factorisation and Adversarial Graph Learning for circRNA-Disease Association Prediction

**DOI:** 10.3390/biology15161427

**Published:** 2026-08-18

**Authors:** Mian-Shuo Lu, Meng-Meng Wei, Chang-Chun Liu, Lei Wang, Cheng-Wei Ruan

**Affiliations:** 1 Guangxi Key Lab of Human-Machine Interaction and Intelligent Decision, Guangxi Academy of Sciences, Nanning 530007, China; 2 School of Computer Science and Technology/School of Artificial Intelligence, China University of Mining and Technology, Xuzhou 221116, China; 3 Department of Anorectal Surgery, The Affiliated Hospital of Xuzhou Medical University, Xuzhou 221006, China

**Keywords:** circRNA-disease association prediction, adversarially regularised variational graph autoencoder, non-negative matrix factorisation, graph representation learning, biological link prediction

## Abstract

Circular RNAs (circRNAs) participate in gene regulation and disease progression, but experimental mapping of circRNA-disease associations (CDAs) remains costly and incomplete. We developed NARVGA, a hybrid prediction framework that combines non-negative matrix factorisation (NMF) with an adversarially regularised variational graph autoencoder (ARVGA). Functional, semantic and Gaussian interaction-profile kernel similarities are integrated; K-means-derived co-membership graphs reduce diffuse similarity connections; ARVGA learns nonlinear topological embeddings; and NMF captures complementary low-rank association patterns. An Extra Trees classifier then scores candidate circRNA-disease pairs. In the original transductive stratified five-fold benchmark on CircR2Disease, NARVGA achieved an area under the receiver operating characteristic curve (AUC) of 0.9868, an area under the precision–recall curve (AUPR) of 0.9887, 94.32% accuracy and a 94.44% F1-score. Ablation analysis identified cluster sparsification as the largest individual contributor, with additional gains from adversarial regularisation and low-rank augmentation. Without task-specific retuning, the model obtained AUCs of 0.9503 on LncRNADisease and 0.9700 on HMDDv4. Performance was stable across GCN depths and cluster settings, and 19 of the 20 highest-ranked hepatocellular carcinoma candidates had supporting published evidence. NARVGA provides a within-network prioritisation tool for RNA-disease association studies, although prospective validation, hard-negative assessment and independent-cohort testing remain necessary.

## Simple Summary

Circular ribonucleic acid molecules help regulate gene activity and may contribute to many diseases. However, laboratory experiments can examine only a small fraction of the possible links between these molecules and diseases, making it difficult to identify the most promising candidates for further study. This study developed a computer-based method that combines several types of biological information to predict likely relationships between circular ribonucleic acids and diseases. When tested on a widely used collection of known associations, the method showed a strong ability to distinguish known associations from unconfirmed ones. It also performed well on two related collections involving other types of ribonucleic acid. In a liver cancer case study, 19 of the 20 highest-ranked circular ribonucleic acids were supported by published studies, while one remained a potentially new candidate. These findings suggest that the method can help researchers select promising associations for laboratory testing, reduce unnecessary experimental screening, and support the discovery of disease-related markers and treatment targets.

## 1. Introduction

Circular RNAs (circRNAs) are covalently closed non-coding transcripts that lack conventional 5′ caps and 3′ poly(A) tails. First observed by Sanger et al. in 1976 [1], circRNAs are now known to be widely expressed and to exhibit tissue-, cell-, and developmental-stage-specific patterns [2]. Their reported functions include microRNA sequestration, protein interaction, regulation of transcription and, for selected transcripts, translation [3].

These properties connect circRNAs to both physiological regulation and disease, particularly cancer [4]. For example, the circRNA-RBCK1 axis has been linked to endothelial dysfunction in heart failure with preserved ejection fraction [5], whereas circ-CCAC1, circ-PTPN22 and circ-ADAMTS6 have been implicated in cholangiocarcinoma-associated invasion, immune escape and angiogenesis [6]. Improved sequencing and curation continue to expand the catalogue of biologically relevant circRNAs [7,8].

Experimental verification remains essential but cannot efficiently screen the large number of possible circRNA-disease pairs. Curated resources such as CircR2Disease and related circRNA databases [9,10,11,12] provide valuable positive associations, yet the association matrix is still sparse and incomplete. Computational models can therefore serve as prioritisation tools by directing laboratory effort towards the most plausible candidates [13,14].

Matrix-factorisation methods address this task by recovering latent structure from the observed association matrix. NMFCDA combines NMF with pseudoinverse learning, KNN-NMF incorporates neighbourhood information, and iCircDA-MF decomposes relational matrices linking circRNAs, genes and diseases [15]. These methods capture global low-rank patterns efficiently, but a purely vector-based representation may not preserve higher-order network topology.

Graph-based models exploit this complementary source of information. VLCDA applies a variational graph autoencoder [16], RGCNCDA incorporates relational graph convolution [17], and MAGCDA and MGRCDA learn multi-hop or metagraph-based representations. Related graph learning strategies have also proved useful for circRNA interaction prediction and biomedical association modelling [18,19]. Nevertheless, dense similarity graphs can propagate uncertain links, and graph embeddings alone may underuse low-rank association structure.

Hybrid approaches have begun to bridge this gap. ELCDA integrates matrix decomposition, GraphSAGE and metapath representations [20], whereas LDA-VGHB combines singular value decomposition with a variational graph autoencoder for lncRNA-disease prediction [21]. These studies motivate a compact framework that explicitly couples linear and nonlinear evidence while controlling graph density and quantifying the contribution of each component.

Relative to VLCDA and GGAECDA, which rely primarily on graph-autoencoder representations, NARVGA adds an explicit low-rank association branch and adversarial latent regularisation. Unlike MAGCDA’s multi-hop attention aggregation, NARVGA first converts similarity profiles into cluster co-membership graphs to control diffuse edges. ELCDA also combines matrix decomposition and graph learning, but uses GraphSAGE and metapath features rather than an adversarial variational encoder and cluster-sparsified similarity graphs [16,20]. The methodological contribution is therefore the evaluated coupling of these mechanisms, rather than the individual use of NMF, graph autoencoding or tree ensembles.

**Figure 1:**
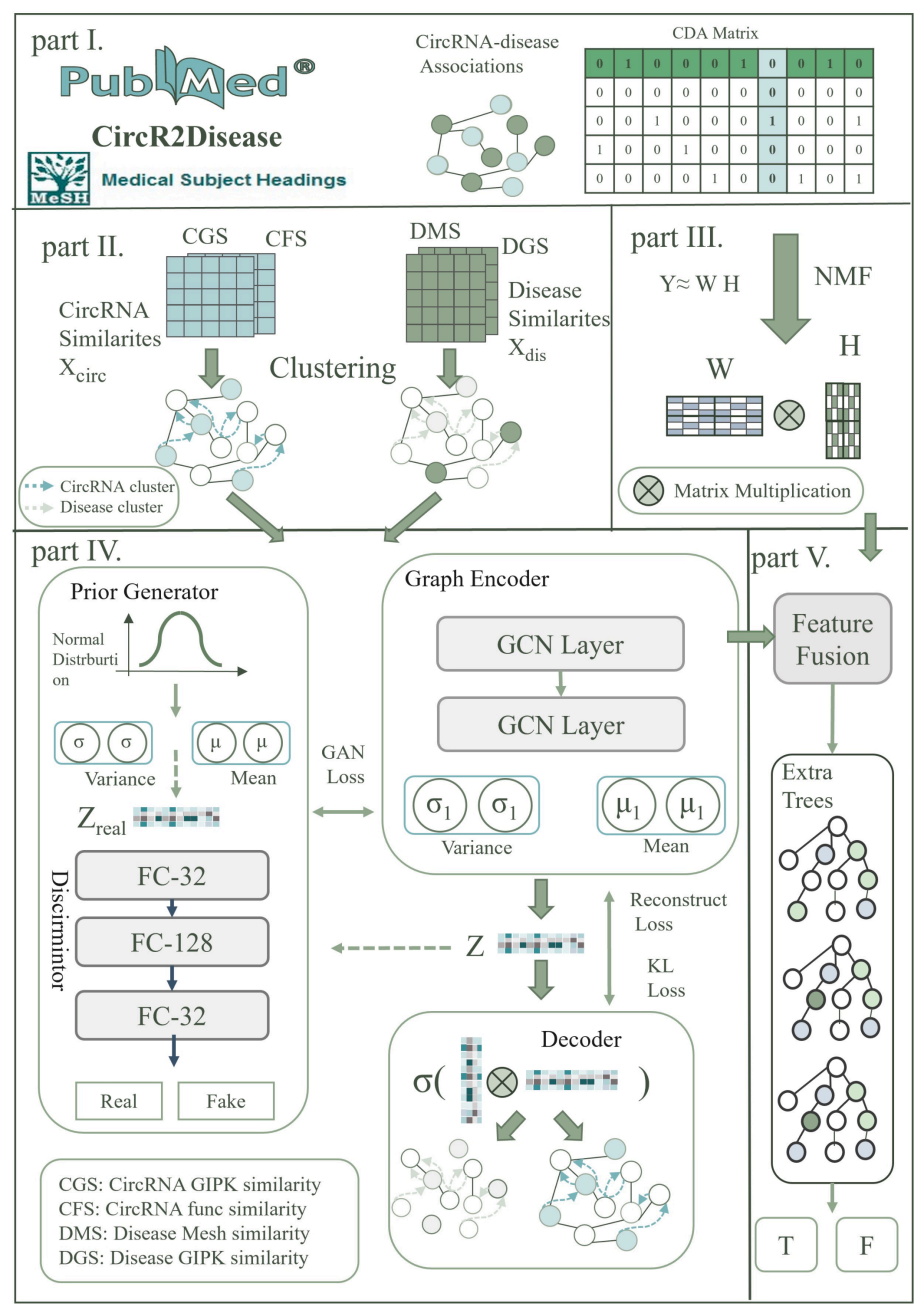
Overview of NARVGA.

To meet this need, we propose NARVGA. K-means clustering first converts integrated circRNA and disease similarity profiles into co-membership graphs, reducing diffuse connections. ARVGA then learns nonlinear graph embeddings under adversarial distributional regularisation, while a parallel NMF branch extracts low-rank linear factors from the CDA matrix. The two representations are concatenated and classified by Extra Trees to generate association probabilities. Figure 1 summarises the complete workflow.

The principal contributions of this study are as follows:

(1) A unified representation combines NMF-derived low-rank factors with ARVGA-derived nonlinear graph embeddings, allowing complementary association and topology information to contribute to each prediction.
(2) Cluster-based graph sparsification limits uncertain similarity connections, while adversarial regularisation constrains the latent distribution and improves representation stability.
(3) A comprehensive evaluation includes stratified five-fold validation, comparison with seven CDA predictors or baselines, mechanism-wise ablation, GCN-depth analysis, cluster-parameter sensitivity and downstream-classifier testing.
(4) Cross-task experiments on lncRNA- and miRNA-disease datasets and a hepatocellular carcinoma case study assess transferability and biological prioritisation beyond the primary benchmark.

## 2. Materials and Methods

### 2.1. Benchmark Datasets

CircR2Disease [16] served as the primary benchmark and contained 607 experimentally supported associations between 561 circRNAs and 100 diseases. For balanced binary evaluation, 607 unlabelled pairs were sampled from entries without recorded associations; these pairs are treated as negatives for evaluation but may include undiscovered positives. Cross-task experiments used LncRNADisease [22] (373 lncRNAs, 252 diseases and 690 positive links) and HMDDv4 [23] (1417 miRNAs, 2304 diseases and 30,476 positive links). Table 1 summarises the resulting matrices.

The cross-task matrices contain lncRNA-disease and miRNA-disease relations rather than circRNA-disease relations; consequently, no circRNA-disease pair can be shared with CircR2Disease, although disease names may overlap. These experiments test transfer across RNA relation types and are not presented as an independent external circRNA cohort.

### 2.2. Similarity Computation

CircRNA GIPK similarity: Each circRNA ci is represented by a binary disease-interaction profile $P\left(\mathrm{ci}\right)$. For circRNA ci, an element equals one when the corresponding association has been verified and zero otherwise. Because circRNAs with related functions are expected to have comparable interaction profiles [24], similarity between ci and cj is evaluated with a Gaussian interaction-profile kernel:

(1) $$\mathrm{CGS}\left(\mathrm{ci},\mathrm{cj}\right)=\exp(-\theta ∥P\left(\mathrm{ci}\right)-P\left(\mathrm{cj}\right)\;{∥}^{2})$$

The kernel-width coefficient θ is obtained from the mean squared magnitude of the n-dimensional interaction profiles:

(2) $$\theta =\frac{1}{n}\sum_{i=1}^{n}{\left|P\left(\mathrm{ci}\right)\right|}^{2}$$

CircRNA functional similarity: Let $\mathrm{CFS}\left(c1,c2\right)$ denote the functional similarity of two circRNAs. Following Wang et al. [25], it is derived from the semantic relatedness of the diseases connected to them. For a disease d and a disease set Dis={d1,d2,…,dn}, similarity between d and that set is the largest pairwise semantic score:

(3) $$SI\left(d,\mathrm{Dis}\right)=\underset{1\leq i\leq n}{\max}\;\mathrm{SI}\left(d,\mathrm{di}\right)$$

Suppose c1 and c2 are linked to Dis1 and Dis2, which contain p and q diseases, respectively. Their functional score $\mathrm{CFS}\left(c1,c2\right)$ averages the semantic evidence obtained in both directions:

(4) $$CFS\left(c1,c2\right)=\frac{\sum_{1\leq i\leq p}SI\left(di,Dis2\right)+\sum_{1\leq j\leq q}SI\left(dj,Dis1\right)}{p+q}$$

Disease MeSH similarity: Disease semantics were obtained from the Medical Subject Headings hierarchy [26]. A disease and its ancestors form a directed acyclic graph (DAG). Terms that occur in fewer disease DAGs are more informative; accordingly, the contribution $U_{d}\left(e\right)$ of term e to disease d is assigned by:

(5) $$U_{d}\left(e\right)=-\log\left(\frac{n_{e}}{N}\right)$$

Here, $n_{e}$ counts the DAGs containing term e, and N is the number of diseases in the collection.

For disease d, summing term-level contributions over its DAG gives the semantic value d:

(6) $$S_{d}=\sum_{e\in N}U_{d}\left(e\right)$$

In this notation, N is the term set in the DAG rooted at d. The similarity of di and dj is then normalised by their total semantic values and calculated over the terms shared by the two DAGs:

(7) $$\mathrm{DMS}\left(\mathrm{di},\mathrm{dj}\right)=\frac{\sum_{e\in \left(N_{\mathrm{di}}\cap N_{\mathrm{dj}}\right)}\left(U_{\mathrm{di}}\left(e\right)+U_{\mathrm{dj}}\left(e\right)\right)}{S_{\mathrm{di}}+S_{\mathrm{dj}}}$$

Disease GIPK similarity: A disease di is likewise encoded by the binary circRNA-interaction profile $V\left(\mathrm{di}\right)$. In $V\left(\mathrm{di}\right)$, each entry records whether the corresponding circRNA is associated with di. Applying the Gaussian kernel to the profiles of di and dj gives [26]:

(8) $$\mathrm{DGS}\left(\mathrm{di},\mathrm{dj}\right)=\exp(-\sigma ∥V\left(di\right)-V\left(dj\right)\;{∥}^{2})$$

The parameter σ controls the kernel scale; profile length is equal to the number of circRNAs.

### 2.3. Similarity Information Fusion

The circRNA similarity used by the model is assembled entry-by-entry. When functional evidence is available for ci and cj, the value in CFS is retained in the integrated matrix CS; otherwise, the corresponding GIPK value from CGS is used:

(9) $$\mathrm{CS}\left(\mathrm{ci},\mathrm{cj}\right)=\left\{\begin{array}{ll} \mathrm{CFS}\;\left(\mathrm{ci},\mathrm{cj}\right), & \mathrm{if}\;\mathrm{CFS}(\mathrm{ci},\mathrm{cj})\;\mathrm{exist}\\ \mathrm{CGS}\;\left(\mathrm{ci},\mathrm{cj}\right), & \mathrm{otherwise} \end{array}\right.$$

The disease matrix DS follows the same rule: an available MeSH semantic score from DMS takes priority, while DGS supplies entries for disease pairs without semantic information:

(10) $$\mathrm{DS}\left(\mathrm{di},\mathrm{dj}\right)=\left\{\begin{array}{ll} \mathrm{DMS}\;\left(\mathrm{di},\mathrm{dj}\right), & \mathrm{if}\;\mathrm{DMS}(\mathrm{di},\mathrm{dj})\;\mathrm{exist}\\ \mathrm{DGS}\;\left(\mathrm{di},\mathrm{dj}\right), & \mathrm{otherwise} \end{array}\right.$$

### 2.4. Linear Feature Extraction

Linear representations are learned by decomposing the association matrix with NMF. Let $M\in R^{m\times n}$ be the non-negative circRNA-by-disease matrix. The approximation of M uses the lower-rank non-negative factors $W\in R^{m\times r}$ and $H\in R^{r\times n}$:

(11) M≈WH

After factorisation, W provides the circRNA-side coordinates, whereas $H^{T}$ provides the disease-side coordinates used in subsequent stages.

### 2.5. Nonlinear Feature Extraction

Nonlinear graph features are obtained with ARVGA [27]. This model couples a variational graph autoencoder with adversarial distribution matching: the encoder captures local and higher-order graph information, while the adversarial objective regularises the latent codes.

#### 2.5.1. Graph Construction

Graph construction begins by embedding nodes in the space of their integrated similarity profiles. K-means assigns each node to the nearest centroid using $L_{2}$ distance; nodes sharing a cluster are connected. The corresponding $L_{2}$ distance between two profiles is:

(12) $$L_{2}\left(a,b\right)=\sqrt{aa^{T}+bb^{T}-2ab^{T}}$$

where a and b are the similarity-profile vectors of the two nodes.

**Figure 2:**
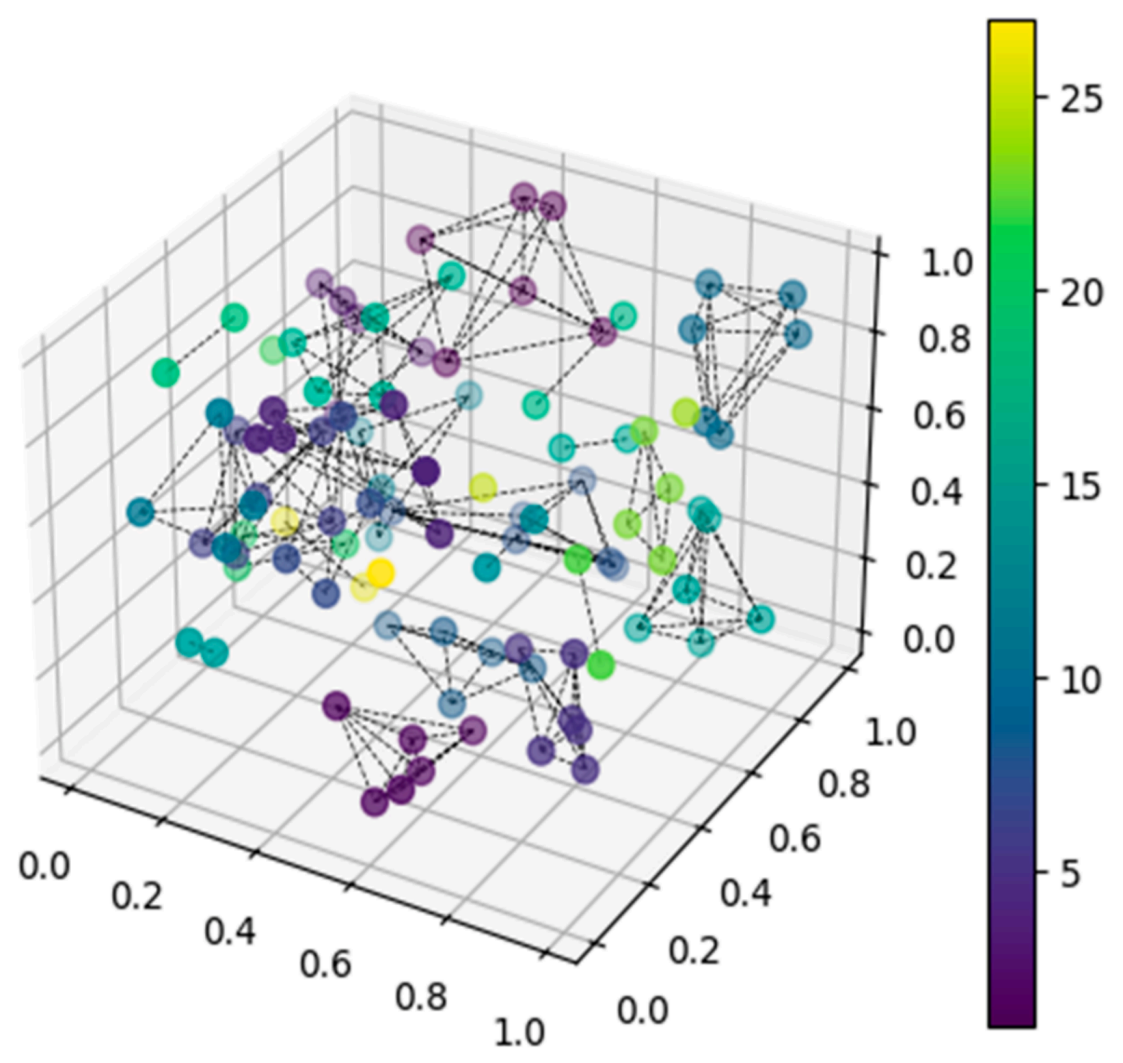
K-means partition of disease nodes used to define graph connectivity.

K-means is applied to the profile space defined by $L_{2}$ distance. The adjacency value is one for a pair in the same cluster and zero otherwise. Figure 2 illustrates the partition obtained for disease nodes. This co-membership matrix becomes A in the graph encoder, while the integrated similarity profiles initialise the node-feature matrix X.

#### 2.5.2. Variational Graph Autoencoder Module

VGAE learns node representations without class labels by combining graph convolution with variational inference. Its latent codes describe neighbourhood structure and dependencies between connected nodes, making the model suitable for the clustered similarity graphs used here.

The encoder first propagates the node attributes through a graph convolutional network (GCN), thereby combining each node’s features with information from its neighbourhood.

Given the adjacency matrix A and initial features $X^{\left(0\right)}$, layer t-th transforms $X^{\left(t\right)}$ into $X^{\left(t+1\right)}$ according to:

(13) Xt+1=ReLU(D~−12AD~−12Xtβt)

In Equation (13), $ReLU\left(\text{·}\right)\;=\;\max\left(0,\;\text{·}\right)$ denotes the activation function. Self-loops are introduced through $\tilde{A}=A+I_{N}$, where $I_{N}$ is the identity matrix. The diagonal degree matrix $\tilde{D}$ is defined by ${\left[\tilde{D}\right]}_{ii}=\sum_{j}{\left[\tilde{A}\right]}_{ij}$. The trainable weights of the GCN layer t-th are denoted by $\beta^{\left(t\right)}$; $X^{\left(t\right)}$ is the layer output and $X^{\left(0\right)}=X$ specifies the input state.

Two graph-convolution heads then parameterise the latent Gaussian distribution through its mean μ and log-variance $\log\sigma^{2}$:

(14) $$\mu ={\mathrm{GCN}}_{\mu }\left(A,X\right)$$

(15) $$\log\sigma^{2}={\mathrm{GCN}}_{\sigma }\left(A,X\right)$$

A latent sample Z is produced with the reparameterisation expression:

(16) $$Z\left(i\right)=\mu_{i}+\sigma_{i}⨀\epsilon ,\;\epsilon ∼N\left(0,I\right)$$

where ϵ is sampled from a standard Gaussian distribution.

The decoder maps the latent matrix Z to an estimate $\hat{A}$ of the graph adjacency:

(17) $$\hat{A}=\sigma \left(ZZ^{T}\right)$$

Here, $\hat{A}$ is the estimated adjacency and Z is the encoder output. Element-wise application of $\sigma \left(\cdot \right)$ maps the inner product $ZZ^{T}$ to [0, 1], providing edge probabilities.

#### 2.5.3. Regularised Adversarial Generation Module

Adversarial regularisation is added because unconstrained graph embeddings may occupy an irregular latent distribution. A discriminator distinguishes samples from the chosen prior from encoder-generated samples, and its feedback encourages smoother, more stable representations.

Prior samples are drawn from N(0,I), while the graph encoder produces latent codes Z. A discriminator with three fully connected layers receives prior and encoded samples and predicts their origin. Its feedback updates the encoder so that generated codes progressively approach the reference distribution.

The clustered circRNA graph is processed by ARVGA to obtain circRNA embeddings. Repeating the same operations on the disease graph yields the disease embeddings used in pair construction.

#### 2.5.4. Loss Function

Training minimises three terms: adjacency reconstruction $L_{\mathrm{recon}}$, variational regularisation $L_{\mathrm{KL}}$ and the adversarial objective $L_{\mathrm{gan}}$.

Reconstruction term ($L_{\mathrm{recon}}$). Binary cross-entropy compares the observed adjacency A with its decoder estimate $\hat{A}$:

(18) $$L_{\mathrm{recon}}=-\sum_{i,j}\left[A_{\mathrm{ij}}\log\left({\hat{A}}_{\mathrm{ij}}\right)+\left(1-A_{\mathrm{ij}}\right)\log\left(1-{\hat{A}}_{\mathrm{ij}}\right)\right]$$

In this expression, $A_{\mathrm{ij}}$ and ${\hat{A}}_{\mathrm{ij}}$ are the observed and reconstructed entries at row i and column j.

Variational term ($L_{\mathrm{KL}}$). The divergence between posterior $q\left(z|x\right)$ and prior $p\left(z\right)$ is measured by:

(19) $$L_{\mathrm{KL}}=-\frac{1}{2}\sum_{i=1}^{N}\sum_{j=1}^{d}\left(1+log\left(\sigma_{ij}^{2}\right)-\mu_{ij}^{2}-\sigma_{ij}^{2}\right)$$

Here, N is the sample count, d is latent dimensionality, and $\mu_{ij}$ and $\sigma_{ij}^{2}$ are the mean and variance associated with code z.

Adversarial term ($L_{\mathrm{gan}}$). The discriminator D is trained against the generator N, which is updated so that samples from N are difficult to distinguish from prior draws:

(20) $$L_{\mathrm{gan}}=-\left(E_{Z∼P\left(Z\right)}\left[\mathrm{logD}\left(Z\right)\right]+E_{\tilde{Z}∼N\left(Z\right)}\left[\log\left(1-D(\tilde{Z})\right)\right]\right)$$

where Z is a latent vector, $P\left(Z\right)$ is the target Gaussian law and $N\left(Z\right)$ denotes the generated distribution.

The final objective adds the reconstruction, variational and adversarial components:

(21) $$L=L_{\mathrm{recon}}+L_{\mathrm{KL}}+L_{\mathrm{gan}}$$

### 2.6. Feature Integration

For each circRNA, the NMF coordinates and ARVGA embedding are concatenated to form a unified entity vector. Disease features are combined in the same way. A candidate circRNA-disease pair is then represented by concatenating the corresponding circRNA and disease vectors before classification.

### 2.7. Extra Trees Classifier for CDA Prediction

Extra Trees [28] converts each integrated pair representation into a CDA probability. The principal analysis used 500 trees, unrestricted depth, square-root feature subsampling and a fixed random state. Averaging the outputs of highly randomised trees reduces variance and limits dependence on any single partition of the feature space.

Each tree is fitted to the training data. At a node, k candidate features are sampled from the full feature set, and a feature is selected using Gini impurity.

Unlike conventional tree ensembles that optimise every candidate threshold, Extra Trees samples split thresholds within the observed feature range. Randomising both feature selection and thresholds increases diversity among trees and can improve generalisation.

### 2.8. Experimental Protocol and Evaluation Metrics

Each dataset was evaluated by stratified five-fold cross-validation. Positive and sampled unlabelled pairs were divided while preserving the class ratio; four folds were used to fit the classifier, and the remaining fold was used for testing, with every fold serving once as the test set. All model variants compared within an experiment used identical folds, and the decision threshold for label-based metrics was 0.5 unless stated otherwise.

Performance was quantified using accuracy (ACC), precision (PRE), recall (REC), specificity (Spec), F1-score and Matthews correlation coefficient (MCC). Threshold-independent discrimination was assessed using AUC and AUPR. Fold-wise values, their means and, where reported, standard deviations were retained. Ablation, depth, cluster-sensitivity and classifier experiments changed only the component under investigation. For cross-task evaluation, the configuration selected on CircR2Disease was applied to LncRNADisease and HMDDv4 without task-specific retuning.

The primary benchmark is transductive: association-derived embeddings were learned from the available benchmark graph before pair-level cross-validation. This protocol measures within-network ranking and is not equivalent to cold-start evaluation of unseen circRNAs or diseases. Random states were fixed for reproducibility, and all models compared within an experiment used identical pair sets and folds. During stratified five-fold cross-validation, fold-specific GIP similarity matrices were generated based on the association information available in the corresponding training fold. The same fold-wise procedure was applied throughout feature construction, model training, and parameter selection, while the held-out associations were reserved for performance evaluation.

NMF used rank 16, random initialisation, and at most 500 iterations; its 16-unit ReLU/sigmoid autoencoder was trained with Adam for 50 epochs and batch size 32. ARVGA used a 16-dimensional latent space, a 1328-unit first hidden layer, a Gaussian prior, Adam with a learning rate of 5 × 10^−4^ and 200 full-batch epochs. Dropout and weight decay were zero, and the reconstruction, KL and adversarial regularisation terms had unit coefficients. The default cluster setting was (Kc, Kd) = (20, 10).

For visual interpretation, out-of-fold probabilities were retained for every evaluation pair. The standardised pair-level representations were clustered by K-means in the full feature space for k = 2–8; k was selected by the silhouette criterion, and principal-component mapping was used only for two-dimensional display. Association labels were not used to construct the clusters.

## 3. Results and Discussion

### 3.1. Performance on CircR2Disease

**Figure 3:**
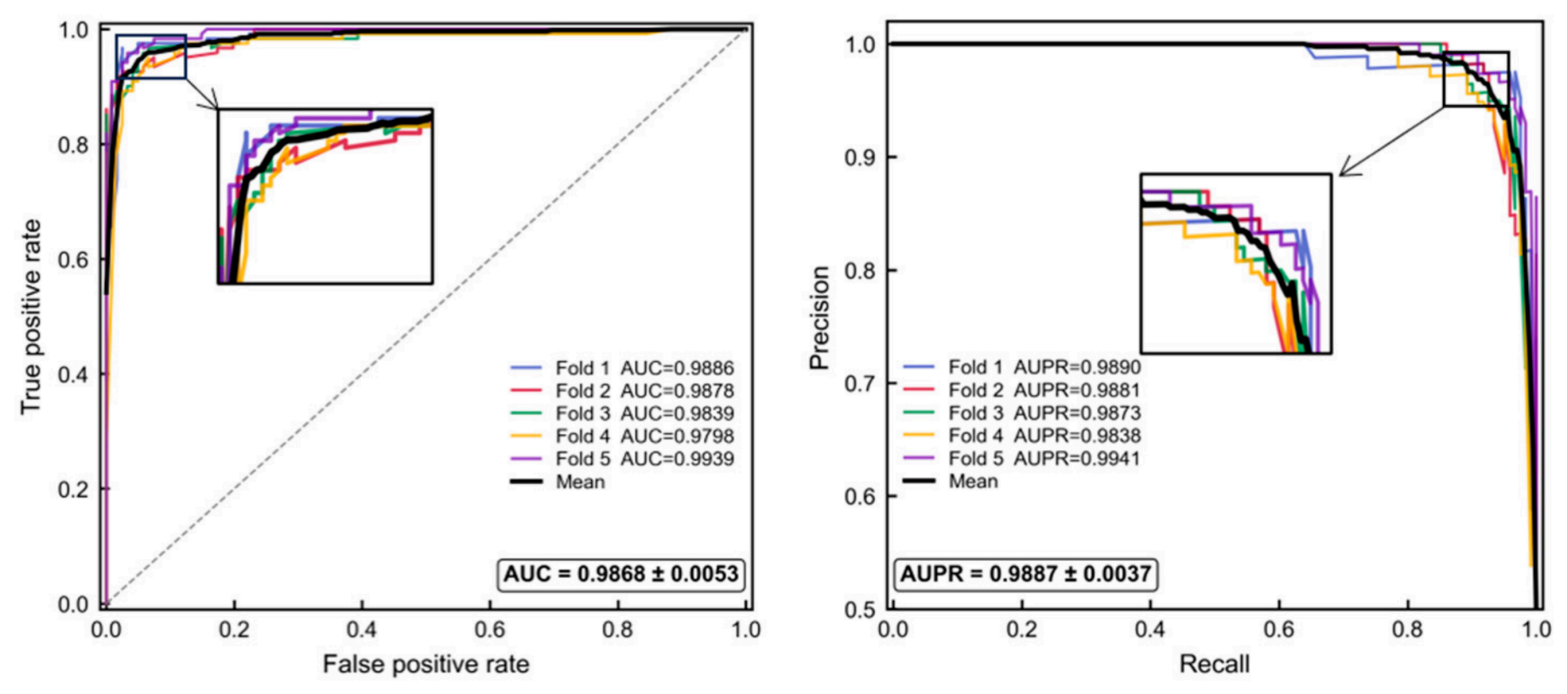
Five-fold ROC (left) and precision–recall (right) curves on CircR2Disease; thin lines denote folds and thick lines denote mean curves.

On CircR2Disease, stratified five-fold cross-validation achieved mean AUC and AUPR values of 0.9868 and 0.9887, respectively (Table 2). The corresponding standard deviations were only 0.0053 and 0.0037, indicating highly consistent probability rankings across folds. At a fixed decision threshold, the model obtained mean ACC and F1 scores of 94.32% and 94.44%, with standard deviations of 1.31 and 1.20 percentage points, respectively. These results show that the strong ranking performance was consistently translated into reliable binary classification. Mean recall reached 96.38%, exceeding both mean precision (92.63%) and specificity (92.26%), suggesting a mild preference for sensitivity. This characteristic is advantageous for preliminary candidate screening, where minimising missed potentially relevant associations is particularly important. The mean MCC was 0.8876, further demonstrating strong agreement between the predicted and observed labels while accounting for all four components of the confusion matrix. The closely clustered ROC and precision–recall curves in Figure 3 visually corroborate the limited fold-to-fold variability reported in Table 2.

### 3.2. Visual Analysis of Prediction Errors and Learned Representations

**Figure 4:**
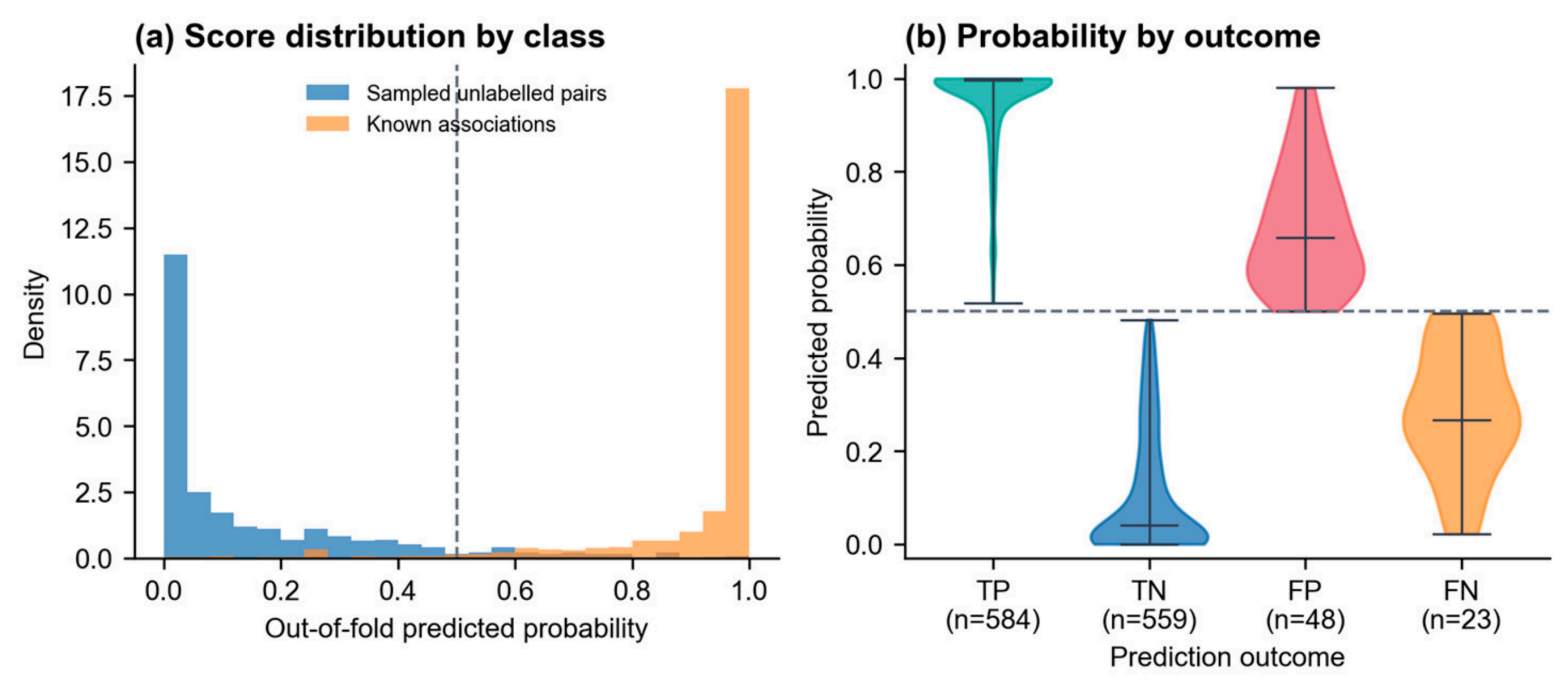
Out-of-fold score distributions on CircR2Disease: (**a**) predicted probabilities for known associations and sampled unlabelled pairs; and (**b**) probabilities grouped by true-positive (TP), true-negative (TN), false-positive (FP) and false-negative (FN) outcomes.

Aggregate metrics do not show how prediction scores are distributed across individual pairs. We therefore examined the out-of-fold probabilities generated by the main configuration (Figure 4). This diagnostic analysis yielded AUC 0.9869, AUPR 0.9884, ACC 94.15% and F1 94.27%, closely matching the fold-mean results in Table 2. Known associations were concentrated near probability 1, whereas sampled unlabelled pairs were concentrated near 0. Among the 1214 evaluated pairs, 48 were false positives, and 23 were false negatives. Their probabilities were closer to the 0.5 decision threshold than those of correctly classified pairs, indicating that most remaining errors occurred in borderline cases rather than through a broad loss of class separation.

**Figure 5:**
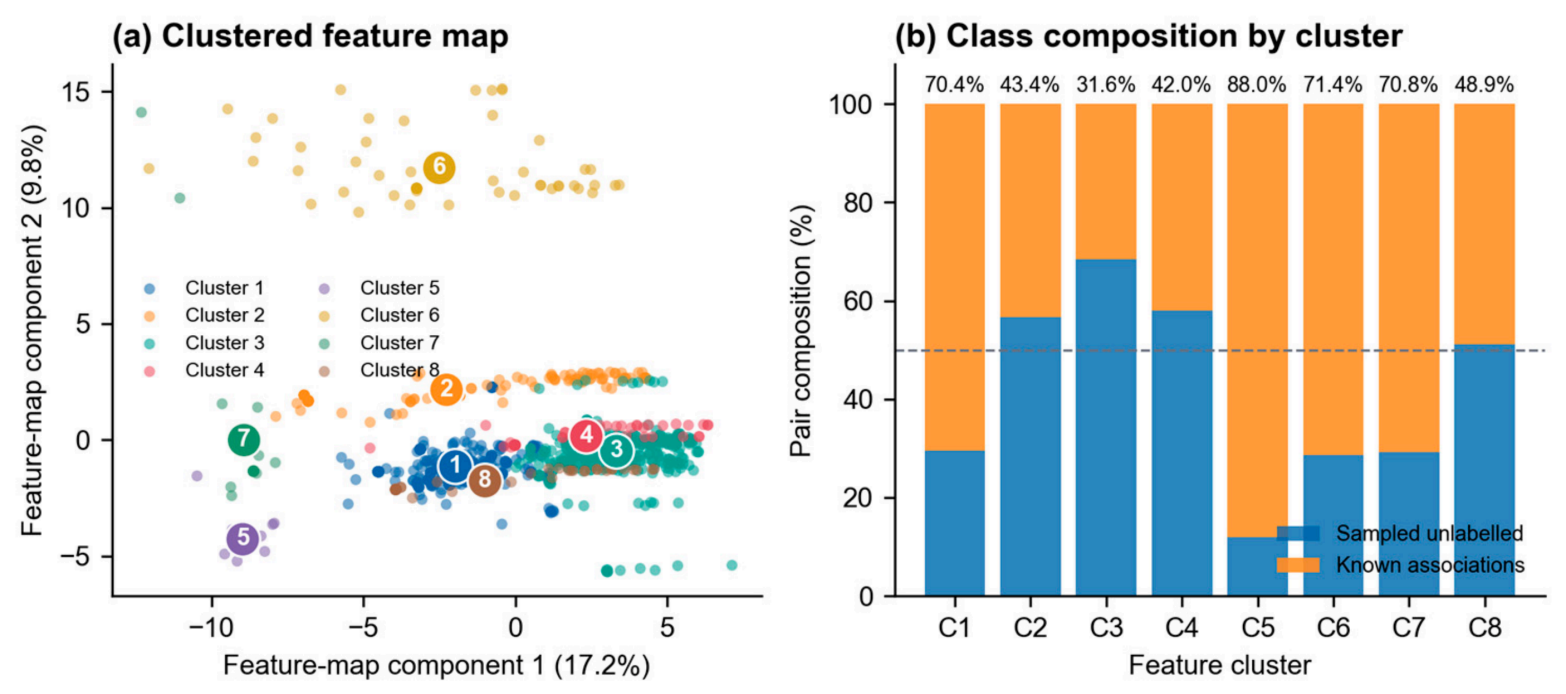
Feature-space organisation of the learned pair representations: (**a**) principal-component map of clusters formed in the full standardised feature space; and (**b**) proportions of known associations and sampled unlabelled pairs within each cluster.

We next examined the organisation of the learned pair representations. K-means selected eight clusters by the silhouette criterion (silhouette score 0.275), and the clusters were displayed using the first two principal components (Figure 5a). Their compositions differed substantially (Figure 5b): Cluster 5 contained 88.0% known associations, whereas Cluster 3 contained 31.6%; Clusters 1, 6 and 7 each contained approximately 70% known associations. Because association labels were not used during clustering, these differences indicate that the hybrid representation organises circRNA-disease pairs into regions with distinct association tendencies. The two-dimensional map is used for qualitative interpretation rather than as evidence of complete class separation.

### 3.3. Comparison with Other Methods

NARVGA was compared with graph-oriented predictors—MAGCDA, MGRCDA, ELCDA [14] and IMS-CDA—and factorisation-oriented methods or baselines, including KNN-NMF, MNMDCDA and ColabFM. AUC, F1 and ACC were compared under compatible five-fold settings. Values for methods without executable implementations were taken from the corresponding benchmark records only when the dataset and validation protocol were compatible.

**Figure 6:**
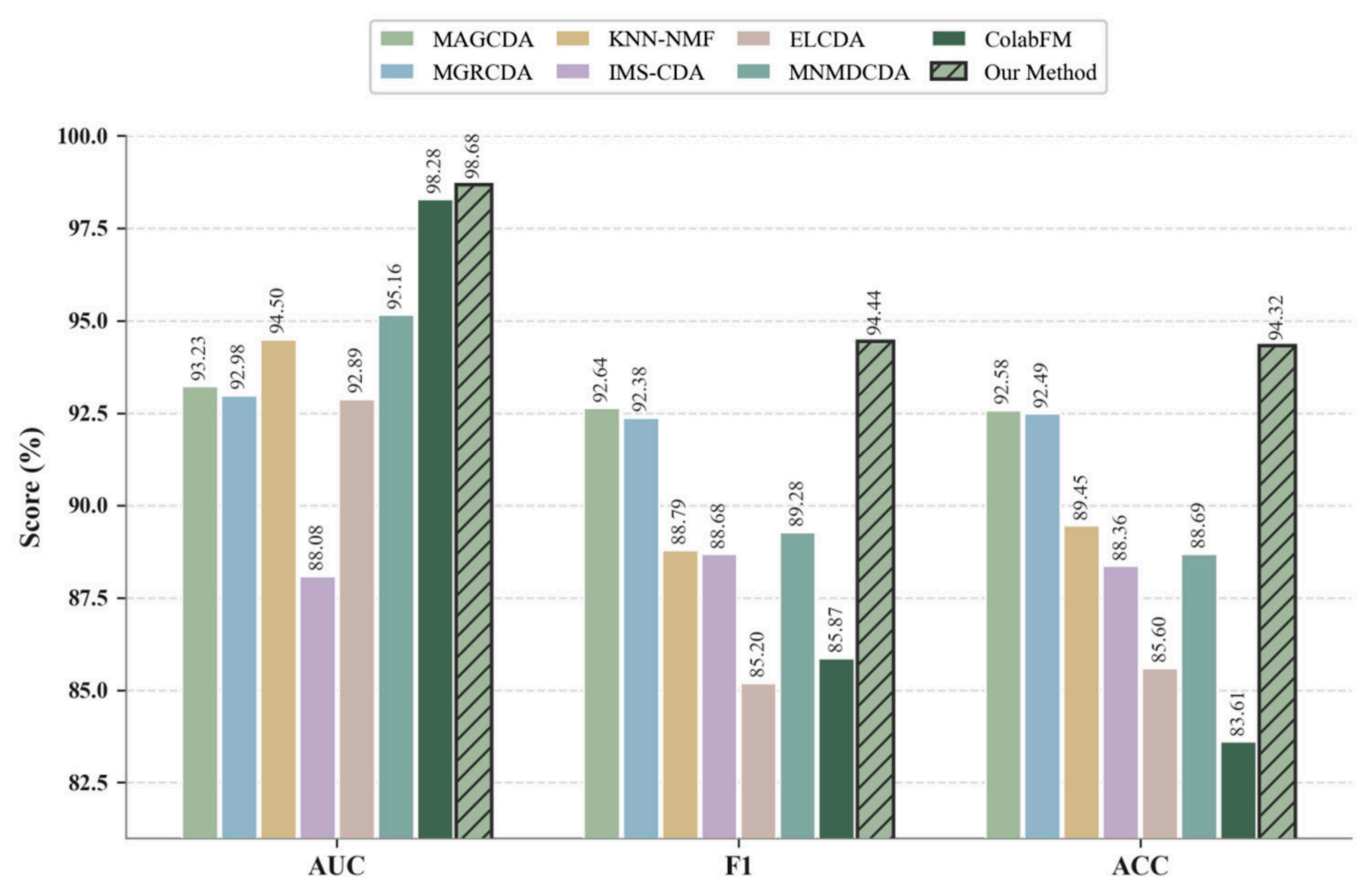
Comparison of AUC, F1 and ACC between NARVGA and seven CDA predictors or benchmark baselines.

NARVGA ranked first for all three reported metrics, achieving AUC 0.9868, F1 94.44% and ACC 94.32% (Figure 6). ColabFM provided the second-highest AUC (0.9828), whereas MAGCDA achieved the next-highest F1 (92.64%) and ACC (92.58%). The result suggests that NARVGA improves both ranking quality and fixed-threshold classification, rather than optimising only one type of measure.

### 3.4. Cumulative Contribution of the Three Complementary Mechanisms

The five configurations were evaluated with identical folds and stopping rules. Starting from a dense graph encoder without cluster sparsification (CS) or adversarial regularisation (AR), AR and CS were activated individually and jointly; low-rank augmentation (LRA) was then added to form the complete model.

**Figure 7:**
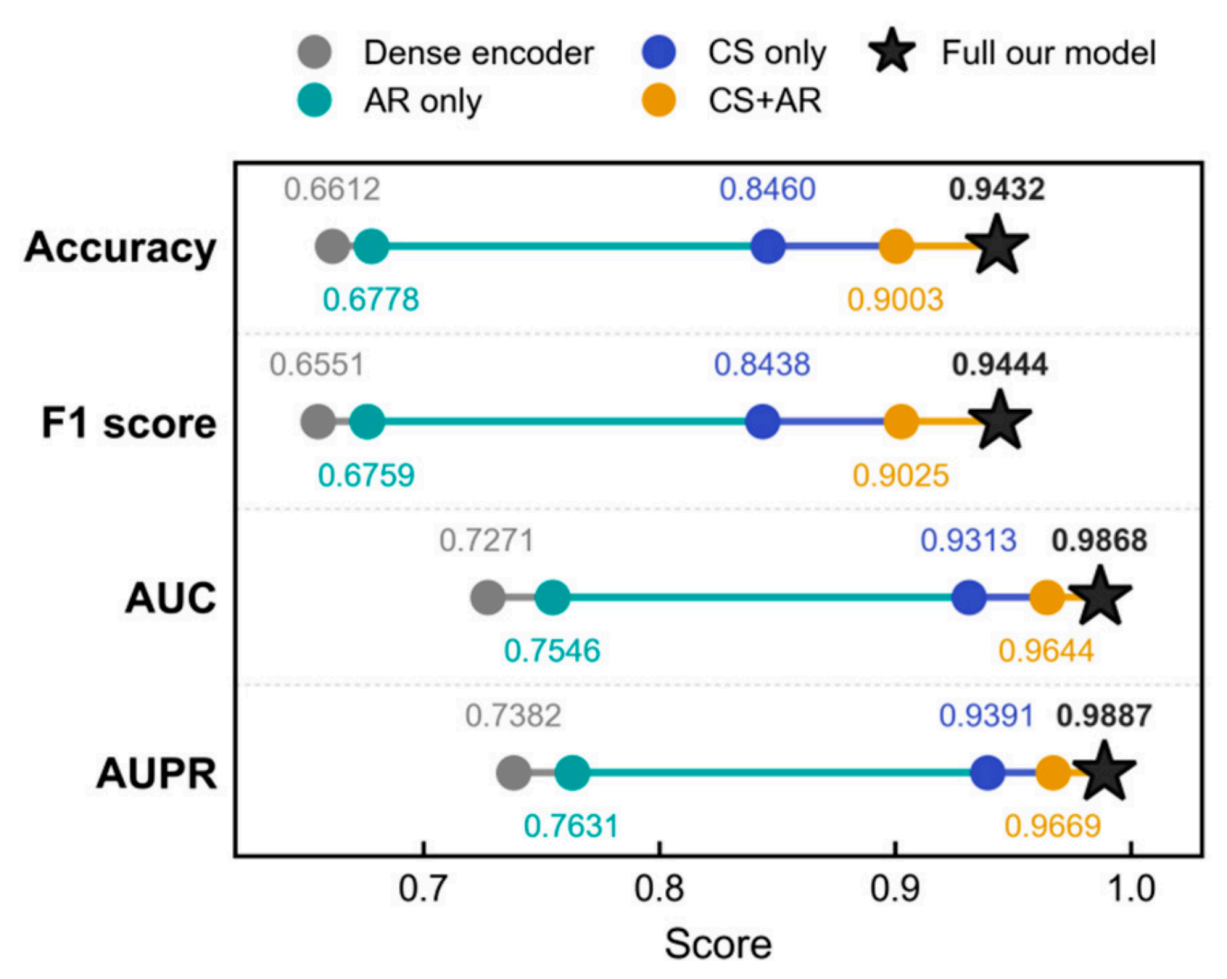
Ablation results for AUC, AUPR, ACC and F1 under five-fold cross-validation.

The dense encoder achieved AUC 0.7271, and AR alone increased it to 0.7546. CS produced the largest individual gain, raising AUC to 0.9313. Combining CS and AR reached 0.9644, showing that adversarial regularisation was most effective after diffuse graph connections had been reduced. Adding LRA increased AUC to 0.9868 and ACC to 94.32%, improvements of 0.0224 AUC and 4.29 percentage points in ACC over CS+AR (Figure 7). Thus, these mechanisms are complementary rather than interchangeable.

### 3.5. Generalisation to ncRNA-Disease Tasks

**Figure 8:**
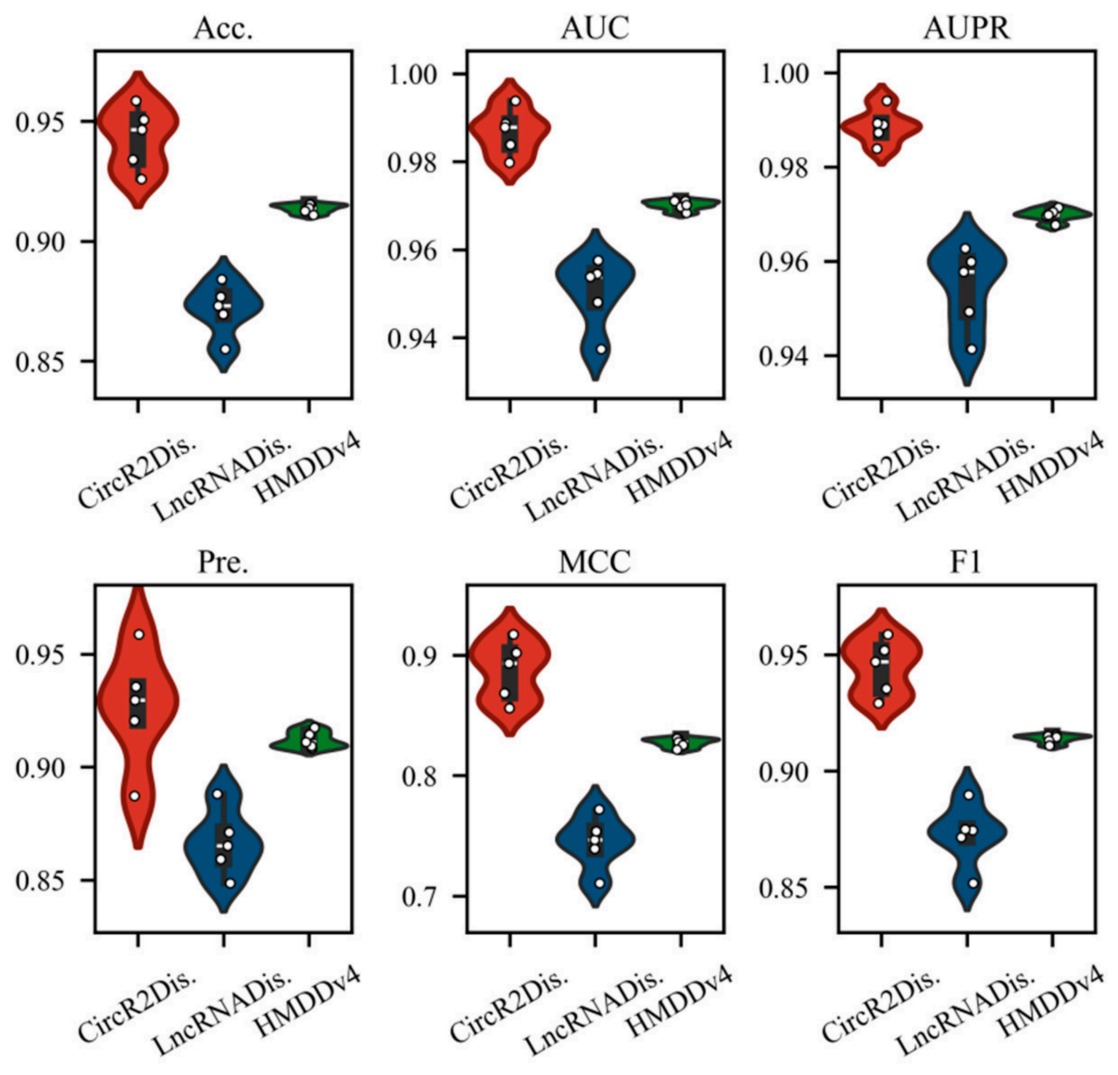
Fold-level distributions of six metrics on CircR2Disease, LncRNADisease and HMDDv4 using an identical configuration.

Cross-task behaviour was assessed without retuning the CircR2Disease configuration. Only the input association matrix and corresponding entity similarities were changed for LncRNADisease and HMDDv4. AUC remained at or above 0.9503 and F1 at or above 87.25% across the three datasets (Table 3 and Figure 8). HMDDv4 showed the narrowest fold dispersion. LncRNADisease produced the lowest ACC (87.17%), while the reduction in AUC was smaller than the reduction in threshold-based metrics; task-specific threshold calibration may therefore recover part of this difference. Because all three datasets concern RNA-disease associations, the experiment supports transfer across RNA classes but does not establish generalisation to unrelated link-prediction domains.

### 3.6. Effect of GCN Encoder Depth

**Figure 9:**
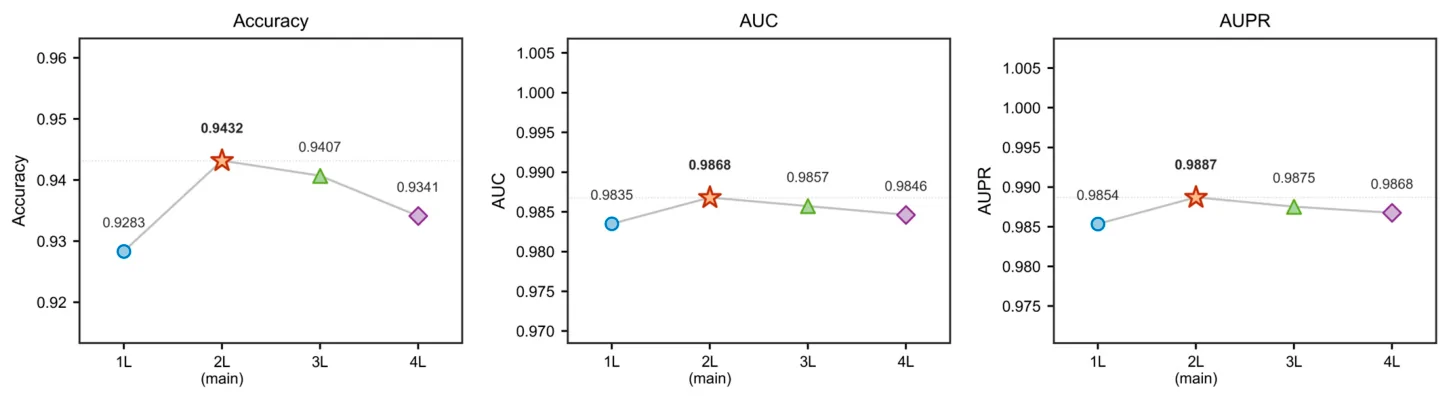
Effect of GCN depth on ACC, AUC and AUPR; the dotted line marks the two-layer default.

The number of GCN encoder layers was varied over L ∈ {1, 2, 3, 4}, while all remaining components and data splits were fixed. Figure 9 reports fold-mean ACC, AUC and AUPR for each depth.

ACC increased from 92.83% at L = 1 to 94.32% at L = 2, then decreased to 94.07% and 93.41% at L = 3 and L = 4, respectively. F1 followed the same trend, whereas AUC and AUPR varied less. The two-layer encoder was therefore retained: it provided the strongest threshold-based results without the additional complexity or possible over-smoothing associated with deeper propagation.

### 3.7. Hyper-Parameter Robustness of Cluster Sparsification

**Figure 10:**
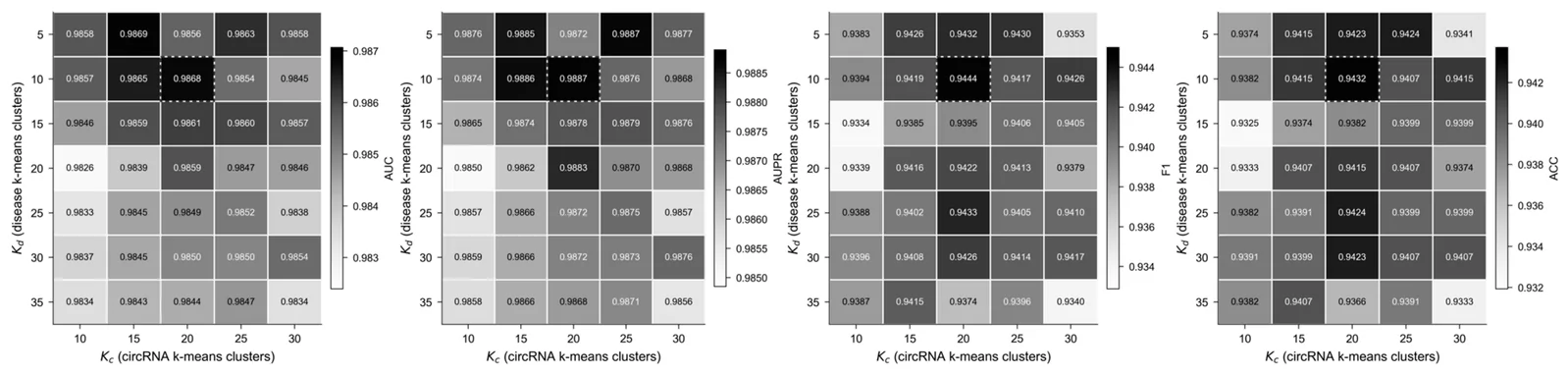
Sensitivity heat maps across the Kc-by-Kd grid.

Cluster sparsification is controlled by the circRNA and disease cluster counts Kc and Kd. We evaluated Kc ∈ {10, 15, 20, 25, 30} and Kd ∈ {5, 10, 15, 20, 25, 30, 35}, producing 35 configurations under the same cross-validation folds (Figure 10 and Table 4).

Variation across this grid was small: standard deviations were below one percentage point for threshold-based metrics and no greater than 0.0011 for AUC or AUPR. Even the least favourable configuration retained AUC above 0.982. The default setting (Kc, Kd) = (20, 10) maximised five of the eight measures; maxima at other settings differed by less than the fold variation observed in the primary experiment. NARVGA is therefore stable in a neighbourhood of the selected cluster counts.

### 3.8. Comparison with Downstream Classifiers

To determine whether the reported performance depended on the prediction head, the learned representations and data splits were fixed while the classifier was changed. Extra Trees, XGBoost, KNN, SVM, DNN, and AdaBoost consequently received identical pair features and train-test partitions.

Extra Trees achieved the strongest overall performance, followed by XGBoost, KNN, SVM, DNN and AdaBoost in the headline comparison (Table 5). All classifiers achieved an AUC above 0.74, but removing cluster sparsification reduced AUC to approximately 0.73 even with the strongest prediction head. This contrast indicates that the quality of the learned representation, rather than the final classifier alone, is central to NARVGA’s performance.

### 3.9. Case Study: Hepatocellular Carcinoma

**Figure 11:**
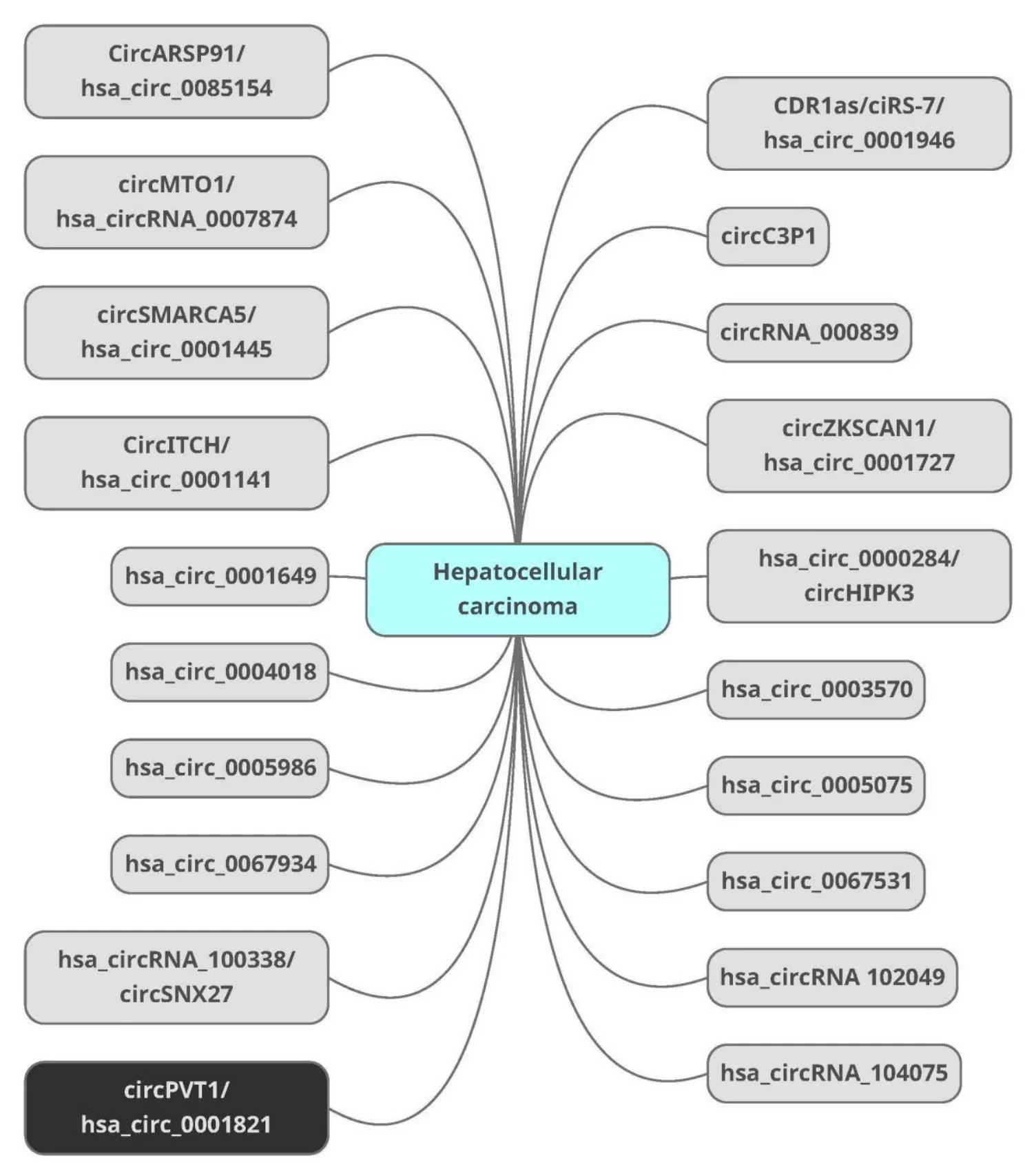
Top 20 predicted HCC-related circRNAs; 19 have supporting experimental evidence.

Hepatocellular carcinoma (HCC), a biologically heterogeneous malignancy with substantial clinical burden [29], was used for disease-specific prioritisation. Database and literature searches provided supporting evidence for 19 of the 20 highest-ranked circRNA candidates; CircPVT1 was the only candidate without identified prior experimental support. Figure 11 distinguishes the supported associations from this testable prediction. The enrichment of previously reported circRNAs near the top of the ranking supports the model’s utility for candidate selection, but it does not replace direct experimental confirmation.

### 3.10. Limitations

Several limitations should be considered when interpreting these results. First, sampled unlabelled pairs are not experimentally verified negatives and may contain genuine but unreported associations. Second, the primary full-graph benchmark is transductive; association-derived features can yield optimistic estimates if interpreted as inductive or cold-start performance. Third, the cross-task datasets contain different RNA relation types and do not constitute an independent external circRNA cohort.

The HCC analysis is retrospective: literature support for highly ranked candidates demonstrates enrichment but not causal validity. Compatible per-candidate score lists from all comparison methods were unavailable for a controlled overlap analysis, and literature-rich circRNAs may be preferentially represented. Future work should test cold-start settings, compare alternative graph-filtering and hard-negative strategies, release baseline candidate scores, and validate novel pairs experimentally.

## 4. Conclusions

NARVGA integrates low-rank association structure with nonlinear graph topology for CDA prediction. Cluster sparsification defines focused neighbourhoods, adversarial training regularises ARVGA embeddings, NMF provides complementary linear factors, and Extra Trees combines the resulting pair features. In the transductive CircR2Disease benchmark, the complete model achieved AUC 0.9868, AUPR 0.9887, ACC 94.32% and F1 94.44%.

The fixed configuration remained effective on LncRNADisease and HMDDv4, was robust to nearby cluster settings and supported HCC candidate prioritisation. These findings position NARVGA as a within-network hypothesis-generation framework rather than a validated cold-start predictor. Independent temporal cohorts, harder-negative designs, alternative graph filtering and prospective wet-laboratory validation are required before practical deployment.

## Figures and Tables

**Table 1:** Datasets used for primary evaluation and cross-task generalisation.

Dataset	RNA-Disease Task	Matrix Size	Positive Links
CircR2Disease	circRNA-disease	561 × 100	607
LncRNADisease	lncRNA-disease	373 × 252	690
HMDDv4	miRNA-disease	1417 × 2304	30,476

**Table 2:** Five-fold cross-validation performance of NARVGA on CircR2Disease.

Fold	AUC	AUPR	ACC (%)	F1 (%)	PRE (%)	REC (%)	Spec (%)	MCC
1	0.9886	0.9890	95.06	95.20	92.97	97.54	92.56	0.9023
2	0.9878	0.9893	93.42	93.55	92.06	95.08	91.74	0.8688
3	0.9839	0.9873	94.65	94.69	93.55	95.87	93.44	0.8933
4	0.9798	0.9839	92.59	92.91	88.72	97.52	87.70	0.8561
5	0.9939	0.9941	95.87	95.87	95.87	95.87	95.87	0.9174
Mean	0.9868	0.9887	94.32	94.44	92.63	96.38	92.26	0.8876
Std	0.0053	0.0037	1.31	1.20	2.60	1.10	2.98	0.0249

**Table 3:** Cross-task generalisation under an identical model configuration.

Dataset	ACC (%)	F1 (%)	AUC	AUPR
CircR2Disease (circRNA)	94.32	94.44	0.9868	0.9887
LncRNADisease (lncRNA)	87.17	87.25	0.9503	0.9542
HMDDv4 (miRNA)	91.36	91.38	0.9700	0.9698

**Table 4:** Summary of the Kc-by-Kd sensitivity analysis.

Metric	Mean	Std	Min	Max	Argmax
F1 (%)	94.01	0.27	93.34	94.44	(20, 10)
ACC (%)	93.93	0.27	93.25	94.32	(20, 10)
AUC	0.9850	0.0011	0.9826	0.9869	(15, 5)
AUPR	0.9871	0.0009	0.9850	0.9887	(20, 10)

**Table 5:** Headline metrics for the downstream classifiers.

Classifier	ACC (%)	F1 (%)	AUC	AUPR
ExtraTrees	94.32	94.44	0.9868	0.9887
XGBoost	91.02	91.37	0.9775	0.9796
KNN	86.57	87.53	0.9365	0.9035
SVM	81.80	82.90	0.8801	0.8004
DNN	77.76	80.11	0.8648	0.8483
AdaBoost	71.17	71.69	0.7474	0.6787

## Data Availability

The public datasets analysed in this study are available from the sources cited in Section 2.1. Processed data, experimental outputs and code are available from the corresponding authors on reasonable request.

## References

[1] Sanger H.L., Klotz G., Riesner D., Gross H.J., Kleinschmidt A.K. (1976). Viroids are single-stranded covalently closed circular RNA molecules existing as highly base-paired rod-like structures. Proc. Natl. Acad. Sci. USA.

[2] Nielsen A.F., Bindereif A., Bozzoni I., Hanan M., Hansen T.B., Irimia M., Kadener S., Kristensen L.S., Legnini I., Morlando M. (2022). Best practice standards for circular RNA research. Nat. Methods.

[3] Goodall G.J., Wickramasinghe V.O. (2021). RNA in cancer. Nat. Rev. Cancer.

[4] Qu L., Yi Z., Shen Y., Lin L., Chen F., Xu Y., Wu Z., Tang H., Zhang X., Tian F. (2022). Circular RNA vaccines against SARS-CoV-2 and emerging variants. Cell.

[5] Li B., Bai W.-W., Guo T., Tang Z.-Y., Jing X.-J., Shan T.-C., Yin S., Li Y., Wang F., Zhu M.-L. (2024). Statins improve cardiac endothelial function to prevent heart failure with preserved ejection fraction through upregulating circRNA-RBCK1. Nat. Commun..

[6] Wen N., Peng D., Xiong X., Liu G., Nie G., Wang Y., Xu J., Wang S., Yang S., Tian Y. (2024). Cholangiocarcinoma combined with biliary obstruction: An exosomal circRNA signature for diagnosis and early recurrence monitoring. Signal Transduct. Target. Ther..

[7] Unlu I., Maguire S., Guan S., Sun Z. (2024). Induro-RT mediated circRNA-sequencing (IMCR-seq) enables comprehensive profiling of full-length and long circular RNAs from low input total RNA. Nucleic Acids Res..

[8] Wei M., Wang L., You Z.-H., Hu P., Zhao B., Huang Z.-A., Huang Y.-A., Yi H. (2026). Dual-channel learning framework for zero-shot CircRNA-miRNA interaction prediction via state space modeling. Proc. AAAI Conf. Artif. Intell..

[9] Fan C., Lei X., Tie J., Zhang Y., Wu F.-X., Pan Y. (2022). CircR2Disease v2.0: An updated web server for experimentally validated circRNA–disease associations and its application. Genom. Proteom. Bioinform..

[10] Yao D., Zhang L., Zheng M., Sun X., Lu Y., Liu P. (2018). Circ2Disease: A manually curated database of experimentally validated circRNAs in human disease. Sci. Rep..

[11] Wu W., Zhao F., Zhang J. (2024). circAtlas 3.0: A gateway to 3 million curated vertebrate circular RNAs based on a standardized nomenclature scheme. Nucleic Acids Res..

[12] Sun Z.-Y., Yang C.-L., Huang L.-J., Mo Z.-C., Zhang K.-N., Fan W.-H., Wang K.-Y., Wu F., Wang J.-G., Meng F.-L. (2024). circRNADisease v2.0: An updated resource for high-quality experimentally supported circRNA-disease associations. Nucleic Acids Res..

[13] Xiao Q., Luo J., Dai J. (2019). Computational prediction of human disease-associated circRNAs based on manifold regularization learning framework. IEEE J. Biomed. Health Inform..

[14] Lu M.-S., Wang L., Wei M.-M., Su X.-R., Zhao B.-W., You Z.-H., Huang D.-S. (2026). LMSCDA: A secondary structure enhanced language model for predicting CircRNA and disease associations. IEEE J. Biomed. Health Inf..

[15] Wei H., Liu B. (2020). iCircDA-MF: Identification of circRNA-disease associations based on matrix factorization. Brief. Bioinform..

[16] Shen S., Qian Y., Zheng J., Liu J., Deng L. Accurately predicting circRNA-disease associations using variational graph auto-encoders and LightGBM. Proceedings of the 2021 IEEE International Conference on Bioinformatics and Biomedicine (BIBM).

[17] Chen Y., Wang Y., Ding Y., Su X., Wang C. (2022). RGCNCDA: Relational graph convolutional network improves circRNA-disease association prediction by incorporating microRNAs. Comput. Biol. Med..

[18] Wei M., Wang L., Su X., Zhao B., You Z. (2025). Multi-hop graph structural modeling for cancer-related circRNA-miRNA interaction prediction. Pattern Recognit..

[19] Li G., Lin Y., Luo J., Xiao Q., Liang C. (2022). GGAECDA: Predicting circRNA-disease associations using graph autoencoder based on graph representation learning. Comput. Biol. Chem..

[20] Yang J., Lei X., Zhang F. (2024). Identification of circRNA-disease associations via multi-model fusion and ensemble learning. J. Cell. Mol. Med..

[21] Peng L., Huang L., Su Q., Tian G., Chen M., Han G. (2024). LDA-VGHB: Identifying potential lncRNA–disease associations with singular value decomposition, variational graph auto-encoder and heterogeneous Newton boosting machine. Brief. Bioinform..

[22] Bao Z., Yang Z., Huang Z., Zhou Y., Cui Q., Dong D. (2019). LncRNADisease 2.0: An updated database of long non-coding RNA-associated diseases. Nucleic Acids Res..

[23] Cui C., Zhong B., Fan R., Cui Q. (2024). HMDD v4.0: A database for experimentally supported human microRNA-disease associations. Nucleic Acids Res..

[24] van Laarhoven T., Nabuurs S.B., Marchiori E. (2011). Gaussian interaction profile kernels for predicting drug-target interaction. Bioinformatics.

[25] Wang D., Wang J., Lu M., Song F., Cui Q. (2010). Inferring the human microRNA functional similarity and functional network based on microRNA-associated diseases. Bioinformatics.

[26] Xiang Z., Qin T., Qin Z.S., He Y. (2013). A genome-wide MeSH-based literature mining system predicts implicit gene-to-gene relationships and networks. BMC Syst. Biol..

[27] Pan S., Hu R., Long G., Jiang J., Yao L., Zhang C. Adversarially regularized graph autoencoder for graph embedding. Proceedings of the Twenty-Seventh International Joint Conference on Artificial Intelligence.

[28] Saeed U., Jan S.U., Lee Y.-D., Koo I. (2021). Fault diagnosis based on extremely randomized trees in wireless sensor networks. Reliab. Eng. Syst. Saf..

[29] Llovet J.M., Castet F., Heikenwalder M., Maini M.K., Mazzaferro V., Pinato D.J., Pikarsky E., Zhu A.X., Finn R.S. (2022). Immunotherapies for hepatocellular carcinoma. Nat. Rev. Clin. Oncol..

